# Acknowledgement of manuscript reviewers 2014

**DOI:** 10.1186/1476-072X-14-6

**Published:** 2015-02-04

**Authors:** Maged N Kamel Boulos

**Affiliations:** Editor-in-Chief, International Journal of Health Geographics, Elgin, Scotland, United Kingdom

## Abstract

The editor of *International Journal of Health Geographics* would like to thank all our reviewers who have contributed to the journal in Volume 13 (2014).

**Philip AbdelMalik**

Canada

**Bahadir Aktug**

France

**Jose Luis Alfonso**

Spain

**Donald Allan**

Australia

**Philippe Apparicio**

Canada

**Mariana Arcaya**

United States of America

**Renato Assuncao**

Brazil

**M. Eric Benbow**

United States of America

**David Berrigan**

United States of America

**John Bethlehem**

Netherlands

**Nevzat Beyazit**

Turkey

**Ling Bian**

United States of America

**Janne Boone-Heinonen**

United States of America

**Francis Boscoe**

Bouvet Island

**Thomas Burgoine**

United Kingdom

**Mattia Calzolari**

Italy

**Anibal Eduardo Carbajo**

Argentina

**Mariângela Carneiro**

Brazil

**Ta-Chien Chan**

Taiwan

**Yang Cheng**

China

**Andrew Clark**

Canada

**Justin Cohen**

United States of America

**Michelle Colacicco-Mayhugh**

United States of America

**Roxanne Connelly**

United Kingdom

**Robert Corner**

Australia

**Robert Cromley**

United States of America

**Andrew Curtis**

United States of America

**Jacqueline Curtis**

United States of America

**Peter Dambach**

Germany

**Lorinne du Toit**

Australia

**Mitch J Duncan**

Australia

**Genevieve Dunton**

United States of America

**Benoit Durand**

France

**Khaled El Emam**

Canada

**Chellafe Ensoy**

Belgium

**Luis E. Escobar**

United States of America

**Daniel Exeter**

New Zealand

**Elisabeth Fichet-Calvet**

Germany

**Rohan Fisher**

Australia

**Edward Fitzgerald**

United States of America

**Robin Flowerdew**

United Kingdom

**Desmond Foley**

United States of America

**Ralph Frerichs**

United States of America

**David Frost**

South Africa

**Claire Fuller**

United Kingdom

**Javier Garcia-Perez**

Spain

**Ramy Ghali**

Egypt

**Daniel Goldberg**

United States of America

**Gordon Gong**

United States of America

**Paul Goodman**

United Kingdom

**Trish Gorely**

United Kingdom

**Estella Graghty**

United States of America

**John Grieco**

United States of America

**Tony Grubesic**

United States of America

**Aline Guttmann**

France

**Michael Hagenlocher**

Austria

**Kristen Hampton**

United States of America

**Anna Hansell**

United Kingdom

**Flo Harrison**

United Kingdom

**Joseph Henderson**

United States of America

**Kevin Henry**

United States of America

**Hans-Werner Hense**

Germany

**Zhiyong Hu**

United States of America

**Zhuojie Huang**

United States of America

**Anke Huss**

Netherlands

**Ezzeldin Ibrahim**

Saudi Arabia

**Monica Jackson**

United States of America

**Bryan James**

United States of America

**Andy Jones**

United Kingdom

**Malia Jones**

United States of America

**Stephen Jones**

United States of America

**Jessica Jones-Smith**

United States of America

**Greg Kearney**

United States of America

**Brian Kelly**

United Kingdom

**Cheryl Kelly**

United States of America

**Louise Kelly-Hope**

United Kingdom

**Jacqueline Kerr**

United States of America

**Yan Kestens**

Canada

**Stefan Kienberger**

Austria

**Gerry Killeen**

Tanzania

**Diane King**

United States of America

**Katherine King**

United States of America

**Charlotte Demant Klinker**

Denmark

**Tom Koch**

Canada

**Daniela Koller**

Germany

**Sari Kovats**

United Kingdom

**Thomas Krafft**

Netherlands

**Martin Kulldorff**

United States of America

**Mei-Po Kwan**

United States of America

**Duncan Lee**

United Kingdom

**Jae Seung Lee**

Korea, South

**Michael Levy**

United States of America

**Angela Liese**

United States of America

**Gina Lovasi**

United States of America

**Stephen Matthews**

United States of America

**Soumya Mazumdar**

Australia

**Robert McCann**

Netherlands

**Matthew McGrail**

Australia

**Julie Méline**

France

**Giulia Melis**

Italy

**Amy Morrison**

Peru

**Stephen Munga**

Kenya

**Sarah-Anne Munoz**

United Kingdom

**Kevin Mwenda**

United States of America

**Kathryn Neckerman**

United States of America

**Robin Nesbitt**

Germany

**Cory Neudorf**

Canada

**John Nuckols**

United States of America

**Isabel Oliver**

United Kingdom

**Dermot O'Reilly**

United Kingdom

**Nicolas Oreskovic**

United States of America

**Cosimo Palagiano**

Italy

**Amber Pearson**

New Zealand

**Maurizio Pisati**

Italy

**Richard G. Prins**

Netherlands

**Wendy Prudhomme O'Meara**

Kenya

**Kevin Ralston**

United Kingdom

**Anne Kerstin Reimers**

Germany

**Bernd Resch**

Germany

**Gregoire Rey**

France

**Alyssa Robinson**

United States of America

**Peter Rogerson**

United States of America

**Gustavo Romero**

Brazil

**Mark Rosenberg**

Canada

**Corrine Ruktanonchai**

United States of America

**Tom Russ**

United Kingdom

**Jose A. Salinas-Perez**

Spain

**Matthew Saltzman**

United States of America

**Jasper Schipperijn**

Denmark

**Jürgen Schweikart**

Germany

**Joseph Sharkey**

United States of America

**Weiqun Shu**

China

**Chantel Sloan**

United States of America

**John Snelgrove**

Canada

**Jeffrey Stanaway**

United States of America

**Aurelia Stefani**

French Guiana

**Rebecca Steinbach**

United Kingdom

**Tim Stinear**

Australia

**Samaah Sullivan**

United States of America

**Guibo Sun**

Hong Kong

**Olivier Telle**

France

**Usa Thisyakorn**

Thailand

**Lukar Thornton**

Australia

**Jie Tian**

United States of America

**Nathan Torbick**

United States of America

**Jelle Van Cauwenberg**

Belgium

**Marie-Claire van der Westhuizen**

South Africa

**Veerle Van Holle**

Belgium

**Edwin van Wijngaarden**

United States of America

**Sophie Vanwambeke**

Belgium

**Teeradache Viangteeravat**

United States of America

**Jean-Francois Viel**

France

**Karen Villanueva**

Australia

**Eloy Viso**

Spain

**John Vulule**

Kenya

**Jeremy Walker**

United Kingdom

**Yvonne Walz**

Germany

**Fahui Wang**

United States of America

**Samuel Wanji**

Cameroon

**Benedict Wheeler**

United Kingdom

**David Wheeler**

United States of America

**Julianne Williams**

United Kingdom

**Ruifu Yang**

China

**Huanming Yang**

China

**Shinya Yasumoto**

Japan

**Nikolaos William Yiannakoulias**

Canada

**Caroline Zeimes**

Belgium

**Shannon Zenk**

United States of America

**Dale Zimmerman**

United States of America

